# Circulating Cell-Free DNA Profiling Predicts the Therapeutic Outcome in Advanced Hepatocellular Carcinoma Patients Treated with Combination Immunotherapy

**DOI:** 10.3390/cancers14143367

**Published:** 2022-07-11

**Authors:** Takayuki Matsumae, Takahiro Kodama, Yuta Myojin, Kazuki Maesaka, Ryotaro Sakamori, Ayako Takuwa, Keiko Oku, Daisuke Motooka, Yoshiyuki Sawai, Masahide Oshita, Tasuku Nakabori, Kazuyoshi Ohkawa, Masanori Miyazaki, Satoshi Tanaka, Eiji Mita, Seiichi Tawara, Takayuki Yakushijin, Yasutoshi Nozaki, Hideki Hagiwara, Yuki Tahata, Ryoko Yamada, Hayato Hikita, Tomohide Tatsumi, Tetsuo Takehara

**Affiliations:** 1 Department of Gastroenterology and Hepatology, Osaka University Graduate School of Medicine, Suita 565-0871, Japan; a0023092@gh.med.osaka-u.ac.jp (T.M.); t-kodama@gh.med.osaka-u.ac.jp (T.K.); myojin@gh.med.osaka-u.ac.jp (Y.M.); k.maesaka@gh.med.osaka-u.ac.jp (K.M.); sakamori@gh.med.osaka-u.ac.jp (R.S.); yuki.tahata@gh.med.osaka-u.ac.jp (Y.T.); ryo726@gh.med.osaka-u.ac.jp (R.Y.); hikita@gh.med.osaka-u.ac.jp (H.H.); tatsumit@gh.med.osaka-u.ac.jp (T.T.); 2 Center for Cancer Research, National Cancer Institute, Bethesda, MD 20892, USA; 3 Genome Information Research Center, Research Institute for Microbial Diseases, Osaka University, Suita 565-0871, Japan; takuwa@ifrec.osaka-u.ac.jp (A.T.); koku@gen-info.osaka-u.ac.jp (K.O.); daisukem@gen-info.osaka-u.ac.jp (D.M.); 4 Department of Gastroenterology and Hepatology, Ikeda Municipal Hospital, Ikeda 563-0025, Japan; yoshiyuki-sawai@hosp.ikeda.osaka.jp (Y.S.); masahide-oshita@hosp.ikeda.osaka.jp (M.O.); 5 Department of Hepatobiliary and Pancreatic Oncology, Osaka International Cancer Institute, Osaka 541-8567, Japan; tasuku.nakabori@oici.jp (T.N.); kazuyoshi.ohkawa@oici.jp (K.O.); 6 Department of Gastroenterology and Hepatology, Osaka Police Hospital, Osaka 543-0035, Japan; mmiya1216@oph.gr.jp; 7 Department of Gastroenterology and Hepatology, National Hospital Organization Osaka National Hospital, Osaka 540-0006, Japan; tanaka.satoshi.eg@mail.hosp.go.jp (S.T.); mita.eiji.zf@mail.hosp.go.jp (E.M.); 8 Department of Gastroenterology and Hepatology, Osaka General Medical Center, Osaka 558-8558, Japan; twr@gh.opho.jp (S.T.); yakushijin@gh.opho.jp (T.Y.); 9 Department of Gastroenterology and Hepatology, Kansai Rosai Hospital, Amagasaki 660-8511, Japan; noza0211@yahoo.co.jp (Y.N.); hagiwara-hideki@kansaih.johas.go.jp (H.H.)

**Keywords:** atezolizumab, bevacizumab, CTNNB1, TERT, HCC, AFP, cfDNA, ctDNA, immunotherapy, biomarker

## Abstract

**Simple Summary:**

Atezolizumab/bevacizumab (Atezo/Bev) combination immunotherapy has become a front-line therapy for unresectable hepatocellular carcinoma (u-HCC), but some patients are initially nonresponders. We investigated the potential of cell-free DNA (cfDNA)/circulating tumor DNA (ctDNA) as biomarkers for predicting the therapeutic outcome of u-HCC patients treated with anti-programmed cell death1-ligand1 (PD-L1)/vascular endothelial growth factor (VEGF) therapy. Patients with high levels of cfDNA showed a significantly lower overall response rate and shorter progression-free survival and overall survival (OS) than those with low levels of cfDNA. Ultradeep sequencing of cfDNA showed that the telomerase reverse transcriptase (TERT) promoter, tumor protein 53 (TP53) and catenin beta 1 (CTNNB1) were the most frequently mutated genes in ctDNA. Lastly, a TERT ctDNA mutation and a high alpha-fetoprotein (AFP) level were independent predictors of shorter OS in u-HCC patients treated with Atezo/Bev therapy and could stratify their prognoses. Collectively, cfDNA/ctDNA profiling may be useful to predict therapeutic outcome in u-HCC patients treated with Atezo/Bev therapy.

**Abstract:**

Combination immunotherapy with anti-programmed cell death1-ligand1 (PD-L1) and anti-vascular endothelial growth factor (VEGF) antibodies has become the standard treatment for patients with unresectable HCC (u-HCC). However, limited patients obtain clinical benefits. Cell-free DNA (cfDNA) in peripheral blood contains circulating tumor DNA (ctDNA) that reflects molecular abnormalities in tumor tissue. We investigated the potential of cfDNA/ctDNA as biomarkers for predicting the therapeutic outcome in u-HCC patients treated with anti-PD-L1/VEGF therapy. We enrolled a multicenter cohort of 85 HCC patients treated with atezolizumab and bevacizumab (Atezo/Bev) between 2020 and 2021. Pretreatment plasma was collected, and cfDNA levels were quantified. Ultradeep sequencing of cfDNA was performed with a custom-made panel for detecting mutations in 25 HCC-related cancer genes. We evaluated the association of cfDNA/ctDNA profiles and clinical outcomes. Patients with high plasma cfDNA levels showed a significantly lower response rate and shorter progression-free survival and overall survival (OS) than those with low cfDNA levels. ctDNA detected in 55% of HCC patients included the telomerase reverse transcriptase (TERT) promoter in 31% of these patients, tumor protein 53 (TP53) in 21%, catenin beta 1 (CTNNB1) in 13% and phosphatase and tensin homolog (PTEN) in 7%. The presence or absence of ctDNA did not predict the efficacy of Atezo/Bev therapy. Twenty-six patients with a TERT mutation had significantly shorter OS than those without. The presence of a TERT mutation and alpha-fetoprotein (AFP) ≥ 400 ng/mL were independent predictors of poor OS according to multivariate Cox proportional hazard analysis and could be used to stratify patients treated with Atezo/Bev therapy based on prognosis. In conclusion, pretreatment cfDNA/ctDNA profiling may be useful for predicting the therapeutic outcome in u-HCC patients treated with anti-PD-L1/VEGF therapy.

## 1. Introduction

Hepatocellular carcinoma (HCC) is a deadly cancer; GLOBOCAN 2020 statistics show that liver cancer is the sixth most common in new cases, and third most common cause of death worldwide [1]. HCC has a high recurrence rate and many patients eventually require systemic therapy. Owing to the great success of multiple recent clinical trials, six systemic therapy regimens are currently available for treating unresectable HCC (u-HCC); these therapies include the use of tyrosine kinase inhibitors, anti-vascular endothelial growth factor receptor 2 (VEGFR2) antibody and combination immunotherapy [2]. Notably, based on the IMbrave150 trial, atezolizumab, anti-programmed cell death1-ligand1 (PD-L1) antibody, and bevacizumab, anti-vascular endothelial growth factor (VEGF) antibody combination immunotherapy (Atezo/Bev) was recently approved and serves as a standard treatment [3]. Atezo/Bev therapy is superior to sorafenib in terms of overall survival (OS), progression-free survival (PFS), and quality of life (QoL) [3]. An update analysis of the IMbrave 150 trial with a median follow-up of 15.6 months showed that the median OS associated with Atezo/Bev therapy was 5.8 months longer than that associated with sorafenib [4]. However, although an objective response was observed in 30% of patients, 19% of treated patients were reported to be nonresponsive [3]. We have also recently reported that approximately 30% of u-HCC cases were initially refractory to Atezo/Bev therapy in a real-world setting [2,5]. Currently, there is no reliable biomarker for predicting HCC patients who will fail to benefit from combination immunotherapy. 

Biomarkers for assessing the efficacy of immune checkpoint inhibitor (ICI) therapies have been actively investigated in many cancer types and include tumor-infiltrating lymphocyte (TIL) counts, intratumor programmed cell death-1 (PD-1)/PD-L1 expression, and tumor mutation burden [6,7]. Regarding HCC, a catenin beta 1 (CTNNB1)-activating mutation is frequently observed and reported to flourish in an immune-desert tumor microenvironment, partly via C-C motif chemokine ligand 5 (CCL5) downregulation [8]. Consequently, Morita M et al. reported that u-HCC patients with Wnt/β-catenin activation and a low degree of CD8+ TILs in tumor tissue showed shorter survival when treated with ICI monotherapy [9]. However, it is unclear whether this association is observed in u-HCC patients treated with Atezo/Bev therapy. Moreover, since u-HCC for whom immunotherapy treatment is recommended are often diagnosed based on imaging studies without tumor biopsy, noninvasive biomarkers are desired for predicting patient outcomes associated with these therapies. 

Cell-free DNA (cfDNA) is released into the blood circulation system from dead cells or by active secretion from live cells. Because cfDNA is easily accessible and noninvasive and has potential utility as a disease biomarker, peripheral blood cfDNA has been investigated in patients with several diseases [10]. In particular, ctDNA, a tiny component of cfDNA, can be detected by next-generation sequencing (NGS) technology and provides information on cancer genome abnormalities without the need for tumor biopsy [11]. This liquid biopsy-based cancer genome profiling strategy is now used in daily practice for personalized cancer therapy, although due to its high medical expense, it is only applied for patients with cancer that is refractory to standard chemotherapy. Circulating tumor DNA (ctDNA) has also been reported to predict tumor burden and treatment response, including response to immunotherapy [12]. However, the potential of cfDNA/ctDNA as an efficacious and/or prognostic biomarker for combination immunotherapy in HCC has never been assessed. Here, we hypothesized that ctDNA may be the surrogate marker of tumor mutation profiles including CTNNB1 mutation, and might predict the response to immunotherapy. We thus performed cfDNA/ctDNA profiling of 85 Atezo/Bev-treated u-HCC patients and evaluated the association of the cfDNA/ctDNA profiling results with clinical outcomes.

## 2. Materials and Methods

### 2.1. Patients and Study Design

A total of 85 unresectable HCC patients who received atezolizumab plus bevacizumab from November 2020 to May 2021 were prospectively enrolled in the Osaka Liver Forum, which includes Osaka University Hospital and 11 other institutions. The inclusion criteria of this biomarker study were as follows. (1) Patients with unresectable hepatocellular carcinoma who are not eligible for locoregional therapy due to local progression or metastasis, (2) a performance status of 0 or 1 according to an Eastern Cooperative Oncology Group (ECOG), (3) Child-Pugh class A or B, and (4) pre-treatment plasma available. The exclusion criteria are as follows. (1) No dynamic contrast-enhanced CT or MRI within 6 months prior to the start of treatment, at the start of treatment, and within 6 to 8 weeks after the treatment initiation, and (2) observation period of less than 6 weeks.

Patients received Atezo/Bev treatment every 3 weeks, and contrast CT or contrast MRI was used to assess treatment response according to the modified Response Evaluation Criteria in Solid Tumors (mRECIST) guidelines [13]. The study protocol was approved by the institutional review board (IRB) committees of Osaka University Hospital and all participating hospitals (IRB No. 921, 19438, 18201). Informed consent was obtained from all patients involved in the study.

We defined Barcelona Clinic Liver Cancer (BCLC) stage classification as follows. BCLC stage A includes a solitary tumor of any size or 2–3 nodules under 3 cm, preserved liver function, performance status 0. BCLC stage B includes multinodular unresectable HCC, preserved liver function, performance status 0. BCLC stage C includes portal invasion, extrahepatic spread, preserved liver function, performance status 1–2. 

### 2.2. Extraction and Quantitative Measurement of cfDNA

Pretreatment blood was collected in Cell-Free DNA BCT^®^CE tubes and shipped immediately to Osaka University Hospital at ambient temperature. Tubes were centrifuged at 2000× *g* for 10 min and the supernatant was collected. Then, the supernatant was centrifuged again at 16,000× *g* for 10 min and the supernatant was collected and preserved at −80 °C. CfDNA was extracted from this supernatant with a MagMAX™Cell-Free DNA Isolation Kit according to the manufacturer’s instructions. The extracted DNA was quantified using a Qubit 1x dsDNA HS Assay Kit (Thermo Fisher Scientific, Waltham, MA, USA). The quality and quantity of cfDNA was assessed with the Cell-Free DNA Screen Tape Assay (Agilent Technologies, Santa Clara, CA, USA). 

### 2.3. Library Preparation, Hybridization Capture and Sequencing of cfDNA

Sequence libraries were prepared using an xGen Prism DNA Library Prep Kit (Integrated DNA Technologies, Coralville, IA, USA) according to the manufacturer’s instructions. A total of 1360 capture probes targeting hotspots and/or entire coding regions of 25 genes, for which recurrent mutations in HCC were previously reported [14], were designed and synthesized. The gene list is provided in Supplementary Table S1. Hybridization capture was then performed according to the IDT protocol ‘xGen hybridization capture of DNA libraries for NGS target enrichment’. Sequencing was performed on an Illumine NovaSeq6000 platform in 101-base paired-end mode.

### 2.4. Variant Analysis

Read mapping was performed according to the analysis guidelines of the xGen Prism DNA Library Kit (Integrated DNA Technologies, Inc.). The unmapped bam (uBAM) was constructed via fastq by using FastqToSam in the Genome Analysis Toolkit (GATK) 4.1.2.0 [15]. Next, Fgbio ExtractUMIsFromBam ver1.4.0 (https://github.com/fulcrumgenomics/fgbio (accessed on 1 June 2021)) was used to extract unique molecular identifiers (UMIs) and add RX tags. The alignment was performed with GATK SamToFastq, Burrows–Wheeler Aligner (BWA) version 0.7.17 [16] and GATK MergeBamAlignment, and the resulting bam was deduplicated by using GATK MarkDuplicates. The overlap between read pairs was eliminated by using Fgbio ClipBam. Variants were called by using GATK Mutect2 and filtered by using FilterMutectCalls and bcftools [17] according to the target region. The resulting vcf files were annotated by using ANNOVAR (24 October 2019) [18]. Variants registered in the Catalog of Somatic Mutations in Cancer (COSMIC) database were included, and single nucleotide polymorphisms (SNPs) registered in the 4.7 kJPN and gnomAD databases were excluded for subsequent analysis of associations with clinical outcomes.

### 2.5. Statistical Analysis

The Mann–Whitney U test was used to evaluate differences between unpaired groups in a nonparametric distribution. For nonparametric multiple comparisons, one-way analysis of variance (ANOVA) followed by the Kruskal–Wallis test was used. For the analysis of categorical data, the chi-square test or Fisher’s exact test was used. Pearson product-moment correlation coefficients were used to assess correlations. For survival analysis, OS was defined as the endpoint from the start of treatment until death from any cause. The Kaplan–Meier and log-rank tests were used to analyzed differences of OS and PFS. Univariate and multivariate Cox proportional hazards regression models were used to identify factors associated with PFS. Statistical significance was *p* values < 0.05. JMP^®^ 13 (SAS Institute Inc. RRID:SCR_014242, Cary, NC, USA) and Prism ver. 8.4.2 for Windows (GraphPad Prism, RRID:SCR_002798, San Diego, CA, USA) were used for analysis.

## 3. Results

### 3.1. Clinical Outcomes of Atezo/Bev Therapy in u-HCC Patients

The clinical characteristics of 85 HCC patients enrolled in this study are shown in Table 1. The median age was 74 years, and 77.6% of the patients were male. The percentage of patients with viral hepatitis was 62.4%. Furthermore, 95.3% of patients had Child–Pugh A, and the median albumin-bilirubin (ALBI) score was −2.35. The numbers of patients diagnosed with BCLC stages A, B, and C were 6, 31, and 48, respectively. The median alpha-fetoprotein (AFP) and des-γ-carboxy prothrombin (DCP) levels were 11 ng/mL and 333 mAU/mL, respectively. Atezo/Bev therapy was initiated as the first-line treatment in 48 patients and as a later-line treatment in 37 patients. The median observation period after the initiation of Atezo/Bev therapy was 286 days.

**Figure 1:**
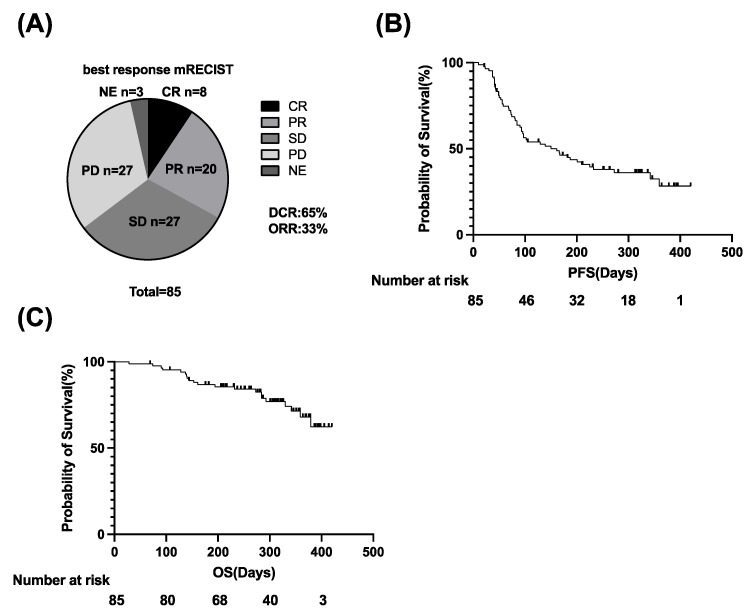
Clinical outcome of Atezo/Bev treatment in 85 u-HCC patients. (**A**) The best response to Atezo/Bev was assessed by mRECIST. (**B**,**C**) Kaplan–Meier curves of progression-free survival (PFS) (**B**) and overall survival (OS) (**C**). u-HCC, unresectable hepatocellular carcinoma; Atezo/Bev, Atezolizumab and bevacizumab; CR, complete response; PR, partial response; SD, stable disease; PD, progressive disease, NE, not evaluated; DCR, disease control rate; ORR, overall response rate.

The overall response rate (ORR) and disease control rate (DCR), evaluated based on mRECIST version 1.1, were 33% and 65%, respectively (Figure 1A). The cumulative PFS rates were 62.5% at 90 days, 44.9% at 180 days, and 36.1% at 270 days. The median PFS was 126 days (Figure 1B). The median OS was not reached, and 21 patients died of HCC (Figure 1C).

### 3.2. Patients with High Plasma cfDNA Levels Show a Significantly Lower ORR and Shorter PFS and OS Than Those with Low cfDNA Levels

**Figure 2:**
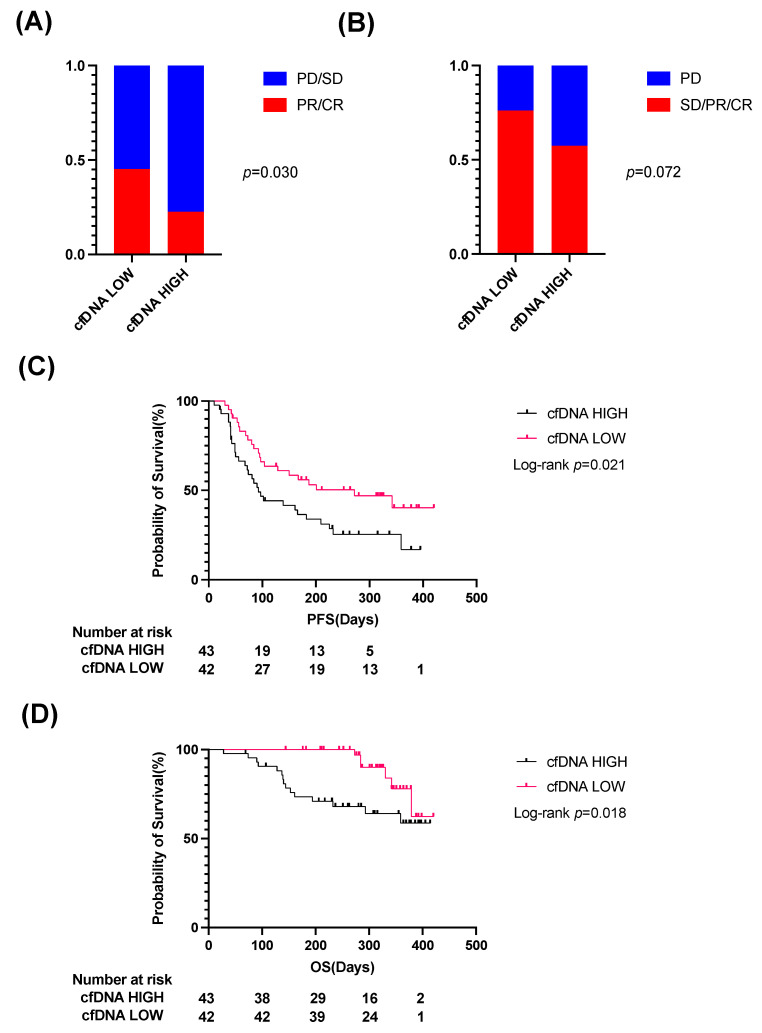
Patients with high plasma cfDNA levels show significantly lower ORR and shorter PFS and OS than those with low cfDNA levels. The baseline cfDNA level was quantified for 85 u-HCC patients treated with Atezo/Bev. The patients were classified into two groups according to the median value of plasma cfDNA level. (**A**,**B**) The best overall response rate (ORR) (**A**) and disease control rate (DCR) (**B**) in each group. (**C**,**D**) The Kaplan–Meier curves of progression-free survival (PFS) (**C**) and overall survival (OS) (**D**) for each group. cfDNA, cell-free DNA; u-HCC, unresectable hepatocellular carcinoma; Atezo/Bev, Atezolizumab and bevacizumab; CR, complete response; PR, partial response; SD, stable disease; PD, progressive disease.

We first evaluated the potential of cfDNA levels to predict the efficacy of Atezo/Bev therapy in u-HCC patients. Pretreatment plasma cfDNA levels were weakly associated with AFP levels and maximal tumor size (Supplementary Figure S1). We then divided patients into two groups based on the median cfDNA levels (cfDNA high vs. cfDNA low). Patients with high cfDNA levels showed significantly higher ALBI scores than those with low cfDNA levels (Supplementary Table S2). Regarding the treatment response to Atezo/Bev therapy, the ORR and DCR were 22.5% and 57.5% in the cfDNA high group and 45.2% and 76.2% in the cfDNA low group, respectively (Figure 2A,B). Patients with high cfDNA levels showed a significantly lower ORR than those with low cfDNA levels (Figure 2A). PFS and OS were also significantly shorter in patients with high cfDNA levels than in patients with low cfDNA levels (Figure 2C,D).

### 3.3. ctDNA Profiling in u-HCC Patients Treated with Atezo/Bev

**Figure 3:**
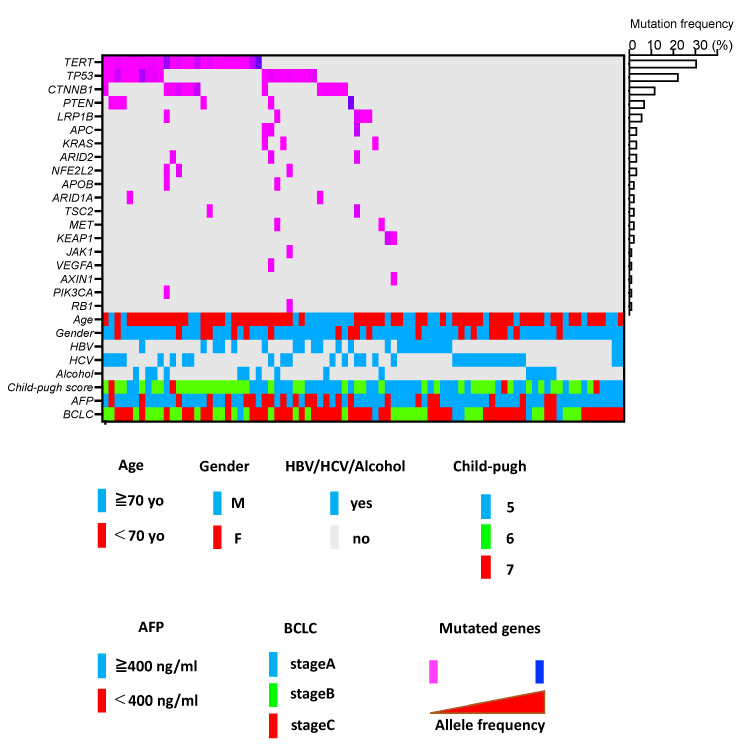
ctDNA profiling of u-HCC patients treated with Atezo/Bev. A heatmap showing the genomic profiling of baseline ctDNA in 85 u-HCC patients treated with Atezo/Bev. Single nucleotide variants are shown in a color scale of variant allele frequency. Genes are listed in the order of mutation frequency. Bottom panel shows age, gender, etiology of background liver disease, Child-Pugh score, AFP level and BCLC stage. ctDNA, circulating tumor DNA; u-HCC, unresectable hepatocellular carcinoma; Atezo/Bev, Atezolizumab and bevacizumab.

To evaluate ctDNA in u-HCC patients treated with Atezo/Bev, we generated a custom panel detecting mutations in 25 genes known to be frequently mutated in HCC (Supplementary Table S1) [14]. Ultradeep sequencing of cfDNA using the custom panel detected ctDNA in 55.3% of patients (Figure 3). We found mutations in 19 out of 25 genes in the panel, and the most frequent mutations were identified in the TERT promoter (31% of these patients), followed by TP53 (22%), CTNNB1 (15%) and PTEN (7%) (Figure 3). The order of mutation frequency in ctDNA was mostly consistent with that previously reported in HCC tumor sites [14], suggesting that ctDNA may reflect genetic abnormalities in HCC tumor tissue. In addition, mutual exclusivity between TP53 and CTNNB1 mutations was also observed in the ctDNA (Figure 3), which is a common mutation pattern in HCC tumor tissue [14,19]. Patients with detectable ctDNA showed a significantly higher neutrophil lymphocyte ratio (NLR) and maximal tumor size and more frequent macrovascular invasion (MVI) than those without detectable ctDNA (Supplementary Table S3). cfDNA levels did not significantly differ between patients with and without detectable ctDNA (Supplementary Figure S2A). The ORR and DCR also did not differ between them (Supplementary Figure S2B,C). Patients with ctDNA showed shorter PFS and OS than those without ctDNA, but these differences were not significant (Supplementary Figure S2D,E). 

### 3.4. Patients with TERT Promoter ctDNA Show Significantly Shorter OS Than Those without

**Figure 4:**
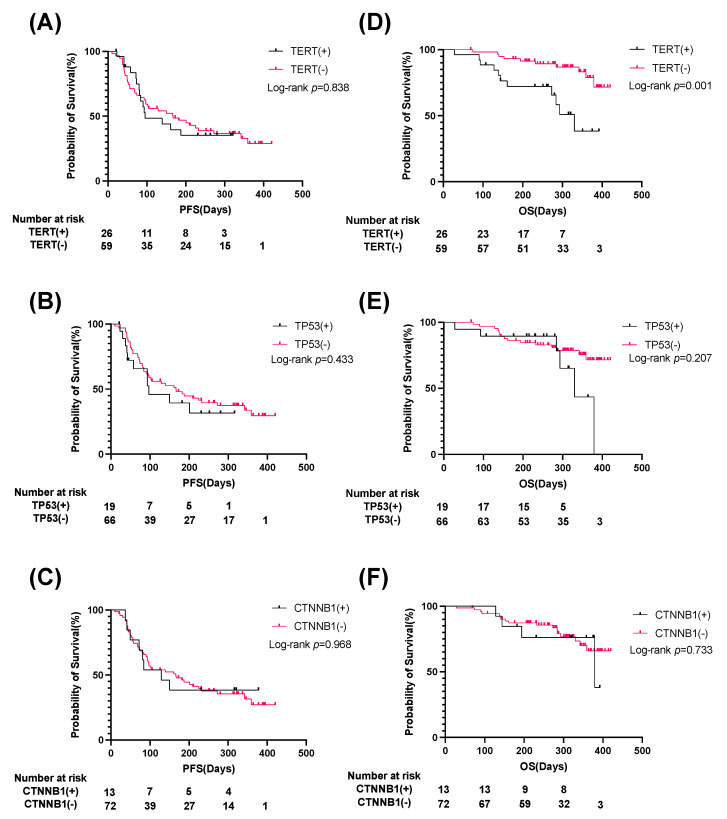
Patients with TERT promoter ctDNA have significantly shorter OS than those without. Total 85 u-HCC patients treated with Atezo/Bev were classified into two groups according to the presence or absence of specific ctDNA mutations including TERT (**A**,**D**), TP53 (**B**,**E**), CTNNB1 (**C**,**F**)**.** The Kaplan–Meier curves of progression-free survival (PFS) (**A**–**C**) and overall survival (OS) (**D**–**F**) for each group. ctDNA, circulating tumor DNA; u-HCC, unresectable hepatocellular carcinoma; Atezo/Bev, Atezolizumab and bevacizumab.

We then assessed the association of frequently mutated genes in cfDNA with clinical outcome. Twenty-six patients with a TERT promoter mutation showed a significantly older age, higher ALBI score, higher Child–Pugh score, higher cfDNA levels, lower prothrombin time (PT) and albumin levels, and less frequent extrahepatic metastasis than patients without a TERT promoter mutation (Supplementary Table S4). Nineteen patients with a TP53 mutation showed significantly higher platelet levels than those without (Supplementary Table S5), while 13 patients with a CTNNB1 mutation showed more frequent MVI and more frequent prior systemic therapy than those without (Supplementary Table S6). The presence or absence of a mutation in any specific gene did not affect clinical outcomes, including therapeutic response and PFS (Supplementary Figure S3A–F, Figure 4A–C). On the other hand, patients with a TERT mutation had significantly shorter OS than those without a TERT mutation (Figure 4D–F). 

### 3.5. TERT ctDNA Mutation and AFP Level Can Be Used to Stratify u-HCC Patients Treated with Combination Immunotherapy Based on Prognosis

Finally, we evaluated the factors associated with shorter PFS and OS. Univariate Cox proportional hazards analysis showed that higher AFP levels, higher NLR, higher intrahepatic tumor number, presence of MVI and prior systemic therapy, and higher cfDNA levels were significantly associated with shorter PFS (Supplementary Table S7). Multivariate Cox proportional hazards analysis showed that MVI and intrahepatic tumor number were independent risk factors for disease progression in Atezo/Bev-treated HCC patients (Supplementary Table S7). Regarding OS, univariate Cox proportional hazards analysis showed that higher AFP and DCP levels, higher NLR, MVI, higher cfDNA levels and TERT ctDNA were significantly associated with shorter OS (Table 2). TERT ctDNA and high AFP levels were independent risk factors for poor prognosis in Atezo/Bev-treated HCC patients by Multivariate Cox proportional hazards analysis (Table 2). 

**Figure 5:**
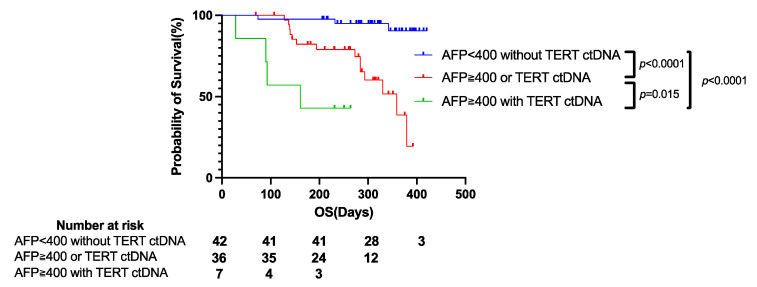
TERT ctDNA mutation and AFP level stratify prognosis of u-HCC patients treated with combination immunotherapy. Total 85 u-HCC patients treated with Atezo/Bev were divided into three groups based on the AFP levels and the presence or absence of TERT ctDNA mutation (AFP levels ≥ 400 ng/mL with TERT ctDNA mutation, AFP levels ≥ 400 ng/mL or TERT ctDNA mutation, AFP levels < 400 ng/mL without TERT ctDNA mutation). The Kaplan–Meier curves of overall survival (OS) for each group. ctDNA, circulating tumor DNA; u-HCC, unresectable hepatocellular carcinoma; Atezo/Bev, Atezolizumab and bevacizumab.

Indeed, when patients were divided into three groups based on AFP levels (AFP ≥ 400 or <400) and the presence or absence of a TERT ctDNA mutation, their prognosis was clearly stratified; patients who had high AFP levels and a TERT ctDNA mutation showed the shortest OS (Figure 5, Supplementary Figure S4). Taken together, these findings suggest that cfDNA/ctDNA monitoring may be useful for predicting the clinical outcome of u-HCC patients treated with combination immunotherapy.

## 4. Discussion

In this study, we evaluated the potential of pretreatment cfDNA/ctDNA profiling as a biomarker to predict the efficacy and/or prognosis of combined anti-PD-L1 and anti-VEGF immunotherapy in u-HCC patients. First, we found that patients with high levels of cfDNA showed a significantly lower ORR and shorter PFS and OS than those with low levels of cfDNA. This suggested the possibility that simple cfDNA quantification may be useful for predicting the clinical outcomes of these patients. Multiple studies have reported the utility of the cfDNA concentration as a prognostic biomarker in a variety of cancer types, including prostate, lung, and breast cancers and neuroblastoma [20,21,22,23,24,25]. Most of these reports show that the cfDNA concentration may reflect the disease stage and thus be positively associated with patient prognosis. The association between high cfDNA concentration and poor chemotherapeutic response has also been reported in breast cancer and ALK^+^ non-small cell lung cancer [26,27]. With regard to HCC, several studies have reported that the cfDNA concentration is higher in patients with HCC than in those with chronic hepatitis and healthy controls [28]. The cfDNA concentration has also been shown to be positively associated with early recurrence and poor OS after surgical resection [29]. On the other hand, the association between cfDNA levels and clinical outcomes of pharmacotherapy has not been well studied in HCC patients. While Nakatsuka T et al. recently reported that posttreatment early changes in cfDNA levels predict the response to molecular targeted agents (MTAs) [30], the current study is the first to show the potential of cfDNA quantification as a therapeutic biomarker of combination immunotherapy in HCC patients. On the other hand, it should be noted that although a high cfDNA level was a significant predictor of OS and PFS by univariate analysis (*p* value 0.024 for OS and 0.023 for PFS), it was not identified as a statistically significant predictor by multivariate analysis (*p* value 0.054 for OS and 0.106 for PFS). Therefore, although we did not find a strong association between cfDNA levels and clinical characteristics (Supplementary Figure S1 and Table S2), the presence of confounding factors should be cautiously considered. Nevertheless, quantification of cfDNA levels in peripheral blood does not require next-generation sequencing or bioinformatics, so cfDNA levels may be a useful and feasible biomarker for predicting the response to immunotherapy in daily clinical practice. Collectively, it is important to conduct further evaluation of its utility in a larger cohort.

We performed ctDNA profiling in 85 u-HCC patients using a custom-made panel detecting mutations in 25 genes that were recurrently mutated in HCC [14]. Ultradeep sequencing of pretreatment cfDNA detected ctDNA in more than half of the patients. Importantly, the three most frequently mutated genes (TERT promoter, TP53 and CTNNB1) are known core HCC driver genes and have been shown to be the most frequently mutated genes in HCC tumor tissues in many large cohorts [14,19,31,32]. Similar mutation profiles have been observed in other studies assessing ctDNA in HCC patients [11,33]. We also identified mutual exclusivity of TP53 and CTNNB1 mutations, which is consistent with previous tumor genome profiling [14]. Taken together, these data supported the validity of our ctDNA profiling.

Regarding treatment response, we first focused on the presence or absence of detectable ctDNA and found a tendency of shorter PFS and OS in patients with detectable ctDNA, but this difference was not significant. The appearance of ctDNA was associated with poor prognostic factors, including the presence of MVI, larger tumor size and higher NLR. We then evaluated the association of specific mutations with treatment response. CTNNB1-mutated HCC has been reported to induce an immune-cold tumor microenvironment (TME) lacking immune cell infiltration and promote resistance to ICI monotherapy [9]. However, neither treatment response (ORR and DCR) nor patient prognosis (PFS and OS) differed between the presence and absence of CTNNB1 mutation. Since we did not perform corresponding tumor genome sequencing in our cohort, it is difficult to reach definite conclusions, but our ctDNA profiling results suggest that a CTNNB1 mutation might not have a large impact on the efficacy of combination immunotherapy in HCC patients. Since anti-VEGF antibody is known to potentiate tumor immunity via normalization of vascular-immune crosstalk [34], it will be interesting in future studies to see whether bevacizumab promotes intratumor immune cell infiltration and changes the CTNNB1-mutated immune-cold TME to an immune-hot TME favorable to atezolizumab therapy.

In addition to CTNNB1, our ctDNA profiling results did not indicate an association of any specific mutation with treatment response and PFS, suggesting that the tumor mutation profile might not affect the efficacy of Atezo/Bev therapy. On the other hand, we found that patients with a TERT mutation showed significantly shorter OS than those without a TERT mutation. Li H et al., have also shown that the presence of TERT mutations was associated with poor prognosis in about 10,000 patients with various types of cancer [35]. Meanwhile, they also showed that the presence of TERT mutations was associated with high TMB score, high neoantigen load, suggesting the greater activity to immunotherapy. Interestingly, they found that prognosis was better in patients with TERT mutation than those without when treated with cytotoxic T-lymphocyte associated antigen 4 (CTLA-4) antibody but was similar in patients with and without TERT mutation when treated with PD-1/PD-L1 antibody. In our cohort, while OS was significantly shorter in patients with TERT mutation than those without, treatment response and PFS were similar in both groups, suggesting that TERT mutation may not affect the response to combined immunotherapy in HCC. On the other hand, while tumor status, including BCLC stage, tumor size, tumor number, and tumor markers (AFP and DCP), did not significantly differ between the presence and absence of a TERT mutation, patients with a TERT promoter mutation had significantly higher Child–Pugh scores and ALBI scores than those without a TERT mutation, suggesting an association between TERT ctDNA and more advanced background liver disease. This could be explained by the fact that TERT promoter mutations occur even in premalignant dysplastic or regenerative nodules in cirrhotic patients [36]. Indeed, TERT ctDNA has been reported to be more frequently found in HCC patients with cirrhosis than in those without [11]. Considering the well-known fact that a background liver functional reservoir is the important determinant of prognosis of u-HCC patients treated with pharmacotherapy [37], the presence of a TERT mutation may reflect more advanced background liver disease and be associated with shorter OS. In addition, our multivariate analysis identified AFP and TERT ctDNA as independent predictors of shorter OS. AFP is a well-known factor reflecting HCC tumor malignancy; thus, u-HCC patients with high AFP levels may have more aggressive tumors, which are associated with shorter OS. Indeed, an association of high AFP levels with shorter OS has been shown in the subgroup analysis of the IMbrave150 trial [4]. Importantly, in the current study, we showed for the first time that the prognosis of u-HCC patients treated with Atezo/Bev therapy was clearly stratified based on TERT ctDNA mutation and AFP level (Figure 5), suggesting the usefulness of ctDNA profiling as a prognostic biomarker for these patients.

The limitations of this study are as follows: (1) it was a retrospective study, (2) there was no racial diversity (all Japanese patients), (3) we did not use a comprehensive cancer gene panel but focused on 25 genes frequently mutated in HCC, and (4) there was no information about tumor mutation burden status or copy number abnormality. We also cannot completely eliminate the possibility of mutation calling error due to clonal hematopoiesis, SNPs, or the sequencer used, because no control sequence for corresponding white blood cells was employed.

In conclusion, we showed the potential usefulness of pretreatment cfDNA/ctDNA profiling for predicting the clinical outcome in u-HCC patients treated with anti-PD-L1 and anti-VEGF combination immunotherapy. To establish noninvasive efficacious and prognostic biomarkers for precision medicine, further external validation in large prospective cohorts will be necessary.

## 5. Conclusions

By analyzing circulating cell-free DNA profiling, we determined that cfDNA/ctDNA profiling is a novel biomarker to predict the prognosis of u-HCC patients treated with anti-PD-L1 and anti-VEGF combination immunotherapy.

## Figures and Tables

**Table 1:** The clinical characteristics of 85 HCC patients enrolled in this study.

Factor	Unit	Value
Age	Years Old	74 (65–80)
Gender	Male/Female	66/19
ECOG PS	0/1	76/9
Etiology	HBV/HCV/HBV + HCV/alcohol/others	22/29/2/15/17
Child-pugh	5/6/7	41/40/4
PT	%	92 (82–102)
ALB	g/dL	3.7 (3.2–3.9)
T-BIL	mg/dL	0.7 (0.5–1.0)
ALBI score		−2.35 (−2.69–−2.02)
ALT	U/L	26 (17–35)
PLT	×10^4^/μL	13.8 (11.2–17.6)
NLR		2.4 (1.8–3.6)
AFP	ng/mL	11 (3–887)
DCP	mAU/mL	333 (65–2614)
Prior systemic therapy	Yes/No	37/48
Extrahepatic metastasis	Yes/No	38/47
Macrovascular invasion	Yes/No	15/70
Maximal tumor size	cm	2.3 (1.6–4.5)
Intrahepatic tumor number	≥5/≤4	36/49
BCLC stage	A/B/C	6/31/48
Observation period	Days	286 (216–359)

Abbreviations: ECOG, Eastern Cooperative Oncology Group; HBV, hepatitis B virus; HCV, hepatitis C virus; PT, prothrombin time; ALB, albumin; T-Bil, total bilirubin; ALBI, Albumin-Bilirubin. ALT, alanine aminotransferase; PLT, platelet; NLR, neutrophil-lymphocyte ratio; AFP, alpha-fetoprotein. DCP, des-γ-carboxy prothrombin; BCLC, Barcelona clinic liver cancer.

**Table 2:** Cox proportional hazards regression model for the prediction of overall survival.

Factor	Unit		Univariate Analysis		Multivariate Analysis	
			Hazard Ratio (95% CI)	*p* Value	Hazard Ratio (95% CI)	*p* Value
Age	Years Old	≥70/<70	0.96 (0.39–2.39)	0.939		
Gender		Male/Female	0.56 (0.23–1.39)	0.212		
ECOG PS		0/1	0.59 (0.17–2.00)	0.398		
Etiology		Viral/non-Viral	1.41 (0.55–3.65)	0.477		
PT	%	≥90/<90	1.12 (0.43–2.90)	0.817		
ALB	g/dL	≥4.0/<4.0	0.95 (0.35–2.60)	0.920		
T-BIL	mg/dL	≥0.7/<0.7	2.26 (0.83–6.18)	0.112		
ALBI score		≥−2.27/<−2.27	1.30 (0.55–3.09)	0.546		
ALT	U/L	≥45/<45	0.62 (0.14–2.66)	0.520		
PLT	×10^4^/μL	≥15/<15	0.47 (0.16–1.39)	0.170		
NLR		≥3.0/<3.0	3.98 (1.62–9.78)	0.003	2.42 (0.80–7.36)	0.119
AFP	ng/mL	≥400/<400	4.79 (1.99–11.54)	0.001	4.90 (1.58–15.13)	0.006
DCP	mAU/mL	≥200/<200	2.96 (1.06–8.25)	0.038	1.72 (0.53–5.57)	0.365
Prior systemic therapy		Yes/No	0.99 (0.42–2.34)	0.984		
Extrahepatic metastasis		Yes/No	0.99 (0.42–2.35)	0.980		
Macrovascular invasion		Yes/No	3.61 (1.49–8.76)	0.005	1.85 (0.62–5.59)	0.273
Intrahepatic tumor number		≥5/≤4	2.29 (0.96–5.46)	0.061		
BCLC stage		A,B/C	0.46 (0.18–1.18)	0.104		
cfDNA	ng/uL	≥2.23/<2.23	2.99 (1.16–7.75)	0.024	2.92 (0.98–8.71)	0.054
TERT		Yes/No	3.93 (1.63–9.44)	0.002	3.25 (1.14–9.28)	0.028

Abbreviations: ECOG, Eastern Cooperative Oncology Group; PT, prothrombin time. ALB, albumin; T-Bil, total bilirubin; ALBI, Albumin-Bilirubin; ALT, alanine aminotransferase. PLT, platelet; NLR, neutrophil-lymphocyte ratio; AFP, alpha-fetoprotein; DCP, des-γ-carboxy prothrombin. BCLC, Barcelona clinic liver cancer; cfDNA, cell-free DNA; TERT, telomerase reverse transcriptase.

## Data Availability

The data presented in this study are available on request from the corresponding author.

## Supplementary Materials

The following supporting information can be downloaded at: https://www.mdpi.com/article/10.3390/cancers14143367/s1, Figure S1: Association between cfDNA levels and clinicopathological variables in 85 u-HCC patients treated with Atezo/Bev, Figure S2: Therapeutic outcomes of 85 u-HCC patients stratified by the presence or absence of ctDNA, Figure S3: Therapeutic outcomes of 85 u-HCC patients stratified by the presence or absence of specific ctDNA mutations, including TERT, TP53, and CTNNB1 mutations, Figure S4: Therapeutic outcomes of 85 u-HCC patients stratified by the presence or absence of TERT mutations and/or AFP levels, Table S1: Gene list in a custom panel for ctDNA detection, Table S2: Clinical characterics of 85 HCC patients stratified by cfDNA levels, Table S3: Clinical characterics of 85 HCC patients stratified by presence or absence of ctDNA. Table S4: Clinical characterics of 85 HCC patients stratified by presence or absence of TERT promoter ctDNA. Table S5: Clinical characterics of 85 HCC patients stratified by presence or absence of TP53 ctDNA. Table S6: Clinical characterics of 85 HCC patients stratified by presence or absence of CTNNB1 ctDNA. Table S7: Cox propotional hazards regression model for the prediction of progression-free survival.
