# Supplementary material for: Circulating Cell-Free DNA Profiling Predicts the Therapeutic Outcome in Advanced Hepatocellular Carcinoma Patients Treated with Combination Immunotherapy
Source: Cancers (Basel). 2022 Jul 11;14(14):3367. doi: 10.3390/cancers14143367 (PMC9320668; doi:10.3390/cancers14143367)
Supplement: Supplementary file 1 [file cancers-14-03367-s001.zip › cancers-1806479-supplementary.pdf]

Supplementary Table S1. Gene list in a custom panel for ctDNA detection

Gene name
ALB
APC
APOB
ARID1A
ARID2
AXIN1
BAP1
CTNNB1
EGFR
FGF19
IL6ST
JAK1
KEAP1
KRAS
LRP1B
MET
NFE2L2
NRAS
PIK3CA
PTEN
RB1
TERT
TP53
TSC2
VEGFA

Supplementary Table S2. Clinical characterics of 85 HCC patients stratified by cfDNA levels

Factor	Unit	cfDNA HIGH	cfDNA LOW	*p* value
Age	Years Old	73(66-81)	75(64-79)	0.790
Gender	Male/Female	30/13	36/6	0.078
ECOG PS	0/1	37/6	39/3	0.308
Etiology	HBV/HCV/HBV+HCV/ alcohol/others	12/15/2/5/9	10/14/0/10/8	0.416
Child-pugh	5/6/7	17/23/3	24/17/1	0.214
PT	%	86(79-100)	92(86-103)	0.391
ALB	g/dL	3.6(3.1-3.9)	3.7(3.4-3.9)	0.054
T-BIL	mg/dL	0.8(0.6-1.2)	0.7(0.5-0.9)	0.065
ALBI score		-2.26(-2.69 - -1.85)	-2.52(-2.72 - -2.15)	0.024
ALT	U/L	31(16-42)	25(17-31)	0.424
PLT	$\times 10^4/\mu L$	13.6(9.4-16.8)	14.1(12.8-19.5)	0.218
NLR		2.4(2.0-3.5)	2.3(1.6-3.7)	0.849
AFP	ng/mL	78(6-3844)	6(3-297)	0.273
DCP	mAU/mL	417(80-3159)	216(60-2793)	0.264
Prior systemic therapy	Yes/No	21/22	16/26	0.318
Extrahepatic metastasis	Yes/No	17/26	21/21	0.332
Macrovascular invasion	Yes/No	8/35	7/35	0.815
Maximal tumor size	cm	2.4(1.7-5.6)	2.0(1.2-4.2)	0.135
Intrahepatic tumor number	≧5/≦4	21/22	15/27	0.221
BCLC stage	A/B/C	3/18/22	3/13/26	0.569

Abbreviations: cfDNA, cell free DNA; ECOG, Eastern Cooperative Oncology Group; HBV, hepatitis B virus HCV, hepatitis C virus; PT, prothrombin time; ALB, albumin; T-Bil, total bilirubin; ALBI, Albumin-Bilirubin ALT, alanine aminotransferase; PLT, platelet; NLR, neutrophil-lymphocyte ratio; AFP, alpha-fetoprotein DCP,des-γ-carboxy prothrombin; BCLC, Barcelona clinic liver cancer

Supplementary Table S3. Clinical characterics of 85 HCC patients stratified by presence or absence of ctDNA

Factor	Unit	ctDNA(+)（N=48）	ctDNA(-)（N=37）	*p* value
Age	Years Old	75(63-80)	72(67-79)	0.827
Gender	Male/Female	37/11	29/8	0.887
ECOG PS	0/1	8/40	1/36	0.038
Etiology	HBV/HCV/HBV+HCV /alcohol/others	13/17/0/10/8	9/12/2/5/9	0.412
Child-pugh	5/6/7	21/25/2	20/15/2	0.571
PT	%	88(82-102)	94(82-104)	0.382
ALB	g/dL	3.6(3.2-3.9)	3.7(3.3-4.0)	0.307
T-BIL	mg/dL	0.7(0.5-1.2)	0.7(0.6-1.0)	0.580
ALBI score		-2.31(-2.67 - -2.02)	-2.43(-2.73 - -2.03)	0.355
ALT	U/L	29(16-35)	25(17-36)	0.903
PLT	$\times 10^4/\mu L$	13.7(10.3-19.2)	14.4(12.4-16.5)	0.354
NLR		3.1(2.0-4.2)	2.0(1.5-2.6)	0.003
AFP	ng/mL	67(4-1796)	7(3-105)	0.932
DCP	mAU/mL	424(96-5342)	127(41-1091)	0.943
Prior systemic therapy	Yes/No	21/27	16/21	0.963
Extrahepatic metastasis	Yes/No	20/28	18/19	0.521
Macrovascular invasion	Yes/No	12/36	3/34	0.043
Maximal tumor size	cm	2.8(1.6-6.2)	1.9(1.5-3.2)	0.011
Intrahepatic tumor number	≧5/≦4	22/26	14/23	0.460
BCLC stage	A/B/C	2/18/28	4/13/20	0.495
cfDNA	ng/uL	2.26(1.20-3.91)	2.22(1.39-3.70)	0.210

Abbreviations: ctDNA, circulating tumor DNA; ECOG, Eastern Cooperative Oncology Group
HBV, hepatitis B virus; HCV, hepatitis C virus; PT, prothrombin time; ALB, albumin; T-Bil, total bilirubin
ALBI, Albumin-Bilirubin; ALT, alanine aminotransferase; PLT, platelet; NLR, neutrophil-lymphocyte ratio
AFP, alpha-fetoprotein; DCP,des-γ-carboxy prothrombin; BCLC, Barcelona clinic liver cancer
cfDNA, cell free DNA

Supplementary Table S4. Clinical characterics of 85 HCC patients stratified by presence or absence of TERT ctDNA mutation

Factor	Unit	TERT(+) (N=26)	TERT(-)(N=59)	*p* value
Age	Years Old	78(71-83)	72(62-78)	0.023
Gender	Male/Female	20/6	46/13	0.915
ECOG PS	0/1	23/3	53/6	0.850
Etiology	HBV/HCV/HBV+HCV /alcohol/others	5/8/0/7/6	17/21/2/8/11	0.456
Child-pugh	5/6/7	6/18/2	35/22/2	0.009
PT	%	83(81-96)	94(84-103)	0.040
ALB	g/dL	3.3(3.1-3.6)	3.8(3.5-4.0)	0.0004
T-BIL	mg/dL	0.8(0.5-1.3)	0.7(0.6-0.9)	0.158
ALBI score		-2.06(-2.39 - -1.76)	-2.52(-2.73 - -2.23)	0.0006
ALT	U/L	31(14-36)	26(17-34)	0.854
PLT	$\times10^4/\mu L$	12.8(8.8-16.7)	14.3(12.6-18.2)	0.228
NLR		2.7(1.7-3.7)	2.3(1.8-3.4)	0.700
AFP	ng/mL	10(3-660)	20(4-981)	0.944
DCP	mAU/mL	759(143-4494)	187(49-1317)	0.784
Prior systemic therapy	Yes/No	11/15	26/33	0.880
Extrahepatic metastasis	Yes/No	7/19	31/28	0.029
Macrovascular invasion	Yes/No	6/20	9/50	0.383
Maximal tumor size	cm	2.4(1.6-5.8)	2.1(1.6-4.3)	0.332
Intrahepatic tumor number	≧5/≦4	15/11	21/38	0.057
BCLC stage	A/B/C	1/13/12	5/18/36	0.209
cfDNA	ng/uL	3.17(2.05-5.58)	1.97(1.11-2.93)	0.020

Abbreviations: TERT,telomerase reverse transcriptase; ctDNA, circulating tumor DNA
ECOG, Eastern Cooperative Oncology Group; HBV, hepatitis B virus; HCV, hepatitis C virus
PT, prothrombin time; ALB, albumin; T-Bil, total bilirubin; ALBI, Albumin-Bilirubin
ALT, alanine aminotransferase; PLT, platelet; NLR, neutrophil-lymphocyte ratio; AFP, alpha-fetoprotein
DCP,des-γ-carboxy prothrombin; BCLC, Barcelona clinic liver cancer; cfDNA, cell free DNA

Supplementary Table S5. Clinical characterics of 85 HCC patients stratified by presence or absence of TP53 ctDNA mutation

Factor	Unit	TP53(+) (N=19)	TP53(-)(N=66)	*p* value
Age	Years Old	75(68-80)	74(64-80)	0.892
Gender	Male/Female	17/2	49/17	0.160
ECOG PS	0/1	16/3	60/6	0.403
Etiology	HBV/HCV/HBV+HCV/ alcohol/others	5/8/0/4/2	17/21/2/11/15	0.682
Child-pugh	5/6/7	9/9/1	32/31/3	0.990
PT	%	91(82-104)	92(82-100)	0.483
ALB	g/dL	3.6(3.1-3.8)	3.7(3.2-4.0)	0.275
T-BIL	mg/dL	0.7(0.4-0.9)	0.7(0.6-1.0)	0.417
ALBI score		-2.36(-2.68 - -1.80)	-2.34(-2.71 - -2.02)	0.518
ALT	U/L	29(19-36)	26(16-35)	0.254
PLT	$\times 10^4/\mu L$	13.8(11.0-23.3)	13.8(11.2-16.3)	0.024
NLR		3.2(2.0-4.4)	2.3(1.8-3.4)	0.153
AFP	ng/mL	88(5-3492)	11(3-297)	0.294
DCP	mAU/mL	759(272-11365)	175(52-1772)	0.378
Prior systemic therapy	Yes/No	10/9	27/39	0.364
Extrahepatic metastasis	Yes/No	6/13	32/34	0.192
Macrovascular invasion	Yes/No	5/14	10/56	0.261
Maximal tumor size	cm	2.4(1.7-7.0)	2.2(1.5-4.5)	0.594
Intrahepatic tumor number	≧5/≦4	11/8	41/25	0.120
BCLC stage	A/B/C	0/9/10	6/22/38	0.274
cfDNA	ng/uL	1.89(0.88-3.11)	2.30(1.40-3.93)	0.287

Abbreviations: TERT,telomerase reverse transcriptase; ctDNA, circulating tumor DNA
ECOG, Eastern Cooperative Oncology Group; HBV, hepatitis B virus; HCV, hepatitis C virus
PT, prothrombin time; ALB, albumin; T-Bil, total bilirubin; ALBI, Albumin-Bilirubin
ALT, alanine aminotransferase; PLT, platelet; NLR, neutrophil-lymphocyte ratio; AFP, alpha-fetoprotein
DCP,des-γ-carboxy prothrombin; BCLC, Barcelona clinic liver cancer; cfDNA, cell free DNA

Supplementary Table S6. Clinical characterics of 85 HCC patients stratified by presence or absence of CTNNB1 ctDNA mutation

Factor	Unit	CTNNB1(+) (N=13)	CTNNB1(-)(N=72)	*p* value
Age	Years Old	69(57-80)	75(68-80)	0.131
Gender	Male/Female	11/2	55/17	0.512
ECOG PS	0/1	13/0	63/9	0.178
Etiology	HBV/HCV/HBV+HCV/ alcohol/others	3/6/0/2/2	19/23/2/13/15	0.867
Child-pugh	5/6/7	6/6/1	35/34/3	0.858
PT	%	87(79-96)	94(82-102)	0.149
ALB	g/dL	3.8(3.1-4.1)	3.7(3.2-3.9)	0.763
T-BIL	mg/dL	0.8(0.5-1.4)	0.7(0.5-1.0)	0.266
ALBI score		-2.32(-2.79 - -1.91)	-2.35(-2.69 - -2.09)	0.991
ALT	U/L	30(14-41)	26(17-35)	0.855
PLT	$\times 10^4/\mu L$	13.4(11.0-15.2)	14.0(11.1-18.4)	0.516
NLR		3.1(1.9-4.2)	2.4(1.8-3.5)	0.568
AFP	ng/mL	5(3-1023)	20(4-757)	0.397
DCP	mAU/mL	197(58-618)	356(66-3824)	0.358
Prior systemic therapy	Yes/No	9/4	28/44	0.042
Extrahepatic metastasis	Yes/No	5/8	33/39	0.623
Macrovascular invasion	Yes/No	5/8	10/62	0.032
Maximal tumor size	cm	2.3(0.8-4.9)	2.3(1.6-4.5)	0.959
Intrahepatic tumor number	≧5/≦4	5/8	31/41	0.758
BCLC stage	A/B/C	0/5/8	6/26/40	0.557
cfDNA	ng/uL	2.35(0.87-6.34)	2.23(1.38-3.59)	0.164

Abbreviations: TERT,telomerase reverse transcriptase; ctDNA, circulating tumor DNA
ECOG, Eastern Cooperative Oncology Group; HBV, hepatitis B virus; HCV, hepatitis C virus
PT, prothrombin time; ALB, albumin; T-Bil, total bilirubin; ALBI, Albumin-Bilirubin
ALT, alanine aminotransferase; PLT, platelet; NLR, neutrophil-lymphocyte ratio; AFP, alpha-fetoprotein
DCP,des-γ-carboxy prothrombin; BCLC, Barcelona clinic liver cancer; cfDNA, cell free DNA

Supplementary Table S7. Cox proportional hazards regression model for the prediction of progression free survival

Factor	Unit		Univariate analysis		Multivariate analysis	
			Hazard ratio(95% CI)	*p* value	Hazard ratio(95% CI)	*p* value
Age	Years Old	≧70/<70	0.60(0.34-1.06)	0.076		
Gender		Male/Female	1.05(0.56-1.97)	0.876		
ECOG PS		0/1	0.95(0.40-2.22)	0.900		
Etiology		Viral/non-Viral	1.18(0.67-2.07)	0.571		
PT	%	≧90/<90	1.23(0.69-2.19)	0.483		
ALB	g/dL	≧4.0/<4.0	1.21(0.65-2.23)	0.547		
T-BIL	mg/dL	≧0.7/<0.7	0.70(0.41-1.21)	0.202		
ALBI score		≧-2.27/<-2.27	0.81(0.47-1.40)	0.812		
ALT	U/L	≧45/<45	1.00(0.47-2.13)	0.998		
PLT	$\times 10^4$/μL	≧15/<15	1.07(0.61-1.90)	0.804		
NLR		≧3.0/<3.0	2.07(1.19-3.59)	0.010	1.78(0.94-3.39)	0.077
AFP	ng/mL	≧400/<400	2.00(1.13-3.55)	0.018	1.59(0.87-2.93)	0.132
DCP	mAU/mL	≧200/<200	1.69(0.96-2.97)	0.069		
Prior systemic therapy		Yes/No	1.83(1.06-3.14)	0.030	1.69(0.96-2.97)	0.068
Extrahepatic metastasis		Yes/No	1.18(0.69-2.03)	0.542		
Macrovascular invasion		Yes/No	3.15(1.70-5.85)	0.0003	2.67(1.36-5.24)	0.004
Intrahepatic tumor number		≧5/≦4	2.75(1.57-4.81)	0.0004	2.70(1.52-4.80)	0.001
BCLC stage		A,B/C	0.60(0.34-1.05)	0.075		
cfDNA	ng/uL	≧2.23/<2.23	1.89(1.09-3.27)	0.023	1.58(0.91-2.77)	0.106
TERT		Yes/No	1.06(0.58-1.95)	0.838		

Abbreviations: ECOG, Eastern Cooperative Oncology Group; PT, prothrombin time
ALB, albumin; T-Bil, total bilirubin; ALBI, Albumin-Bilirubin; ALT, alanine aminotransferase
PLT, platelet; NLR, neutrophil-lymphocyte ratio; AFP, alpha-fetoprotein; DCP, des-γ-carboxy prothrombin
BCLC, Barcelona clinic liver cancer; cfDNA, cell free DNA; TERT,telomerase reverse transcriptase

Supplementary Figure Legends

Supplementary Figure S1. Association between cfDNA levels and clinicopathological variables in 85 u-HCC patients treated with Atezo/Bev. cfDNA, cell-free DNA; u-HCC, unresectable hepatocellular carcinoma; Atezo/Bev, atezolizumab and bevacizumab.

Supplementary Figure S2. A total of 85 u-HCC patients treated with Atezo/Bev were divided into 2 groups by the presence or absence of ctDNA. **(A)** cfDNA levels in each group. (**B-C**) The best overall response rate (ORR) **(B)** and disease control rate (DCR) **(C)** in each group. **(C-D)** The Kaplan–Meier curves of progression-free survival (PFS) **(C)** and overall survival (OS) **(D)** for each group. ctDNA, circulating tumor DNA; u-HCC, unresectable hepatocellular carcinoma; Atezo/Bev, atezolizumab and bevacizumab; CR, complete response; PR, partial response; SD, stable disease; PD, progressing disease.

Supplementary Figure S3. A total of 85 u-HCC patients treated with Atezo/Bev were divided into 2 groups based on the presence or absence of specific ctDNA mutations, including TERT **(A, D)**, TP53 **(B, E)**, and CTNNB1 **(C, F)** mutations**.** The best overall response rate (ORR) **(A-C)** and disease control rate (DCR) **(D-F)** in each group. ctDNA, circulating tumor DNA; u-

HCC, unresectable hepatocellular carcinoma; Atezo/Bev, atezolizumab and bevacizumab.

Supplementary Figure S4. A total 85 u-HCC patients treated with Atezo/Bev were divided into 4 groups based on the AFP levels and/or the presence or absence of TERT ctDNA mutation. The Kaplan–Meier curves of overall survival (OS) for each group. ctDNA, circulating tumor DNA; u-HCC, unresectable hepatocellular carcinoma; Atezo/Bev, Atezolizumab and bevacizumab.

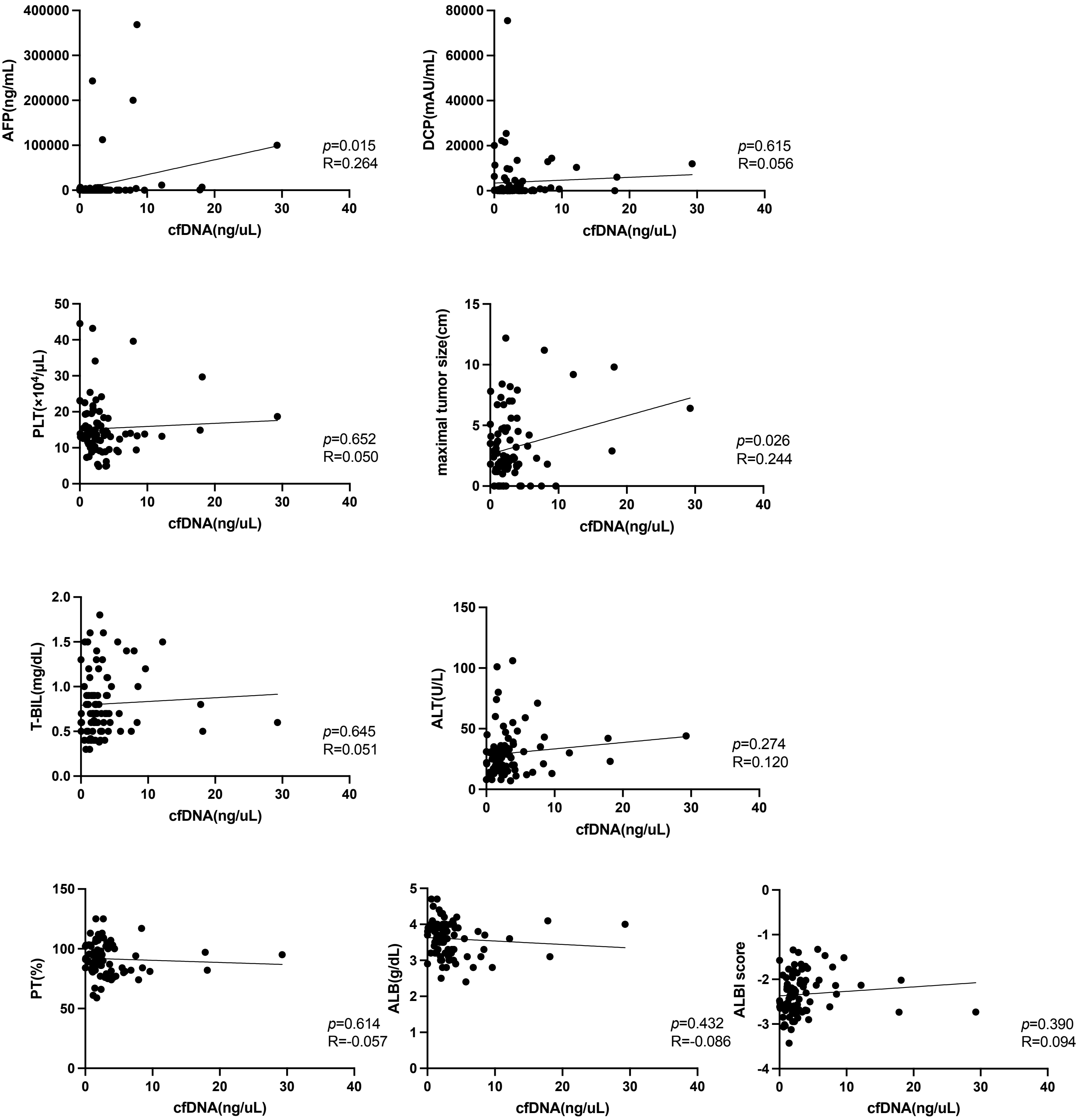

Supplementary Figure S1

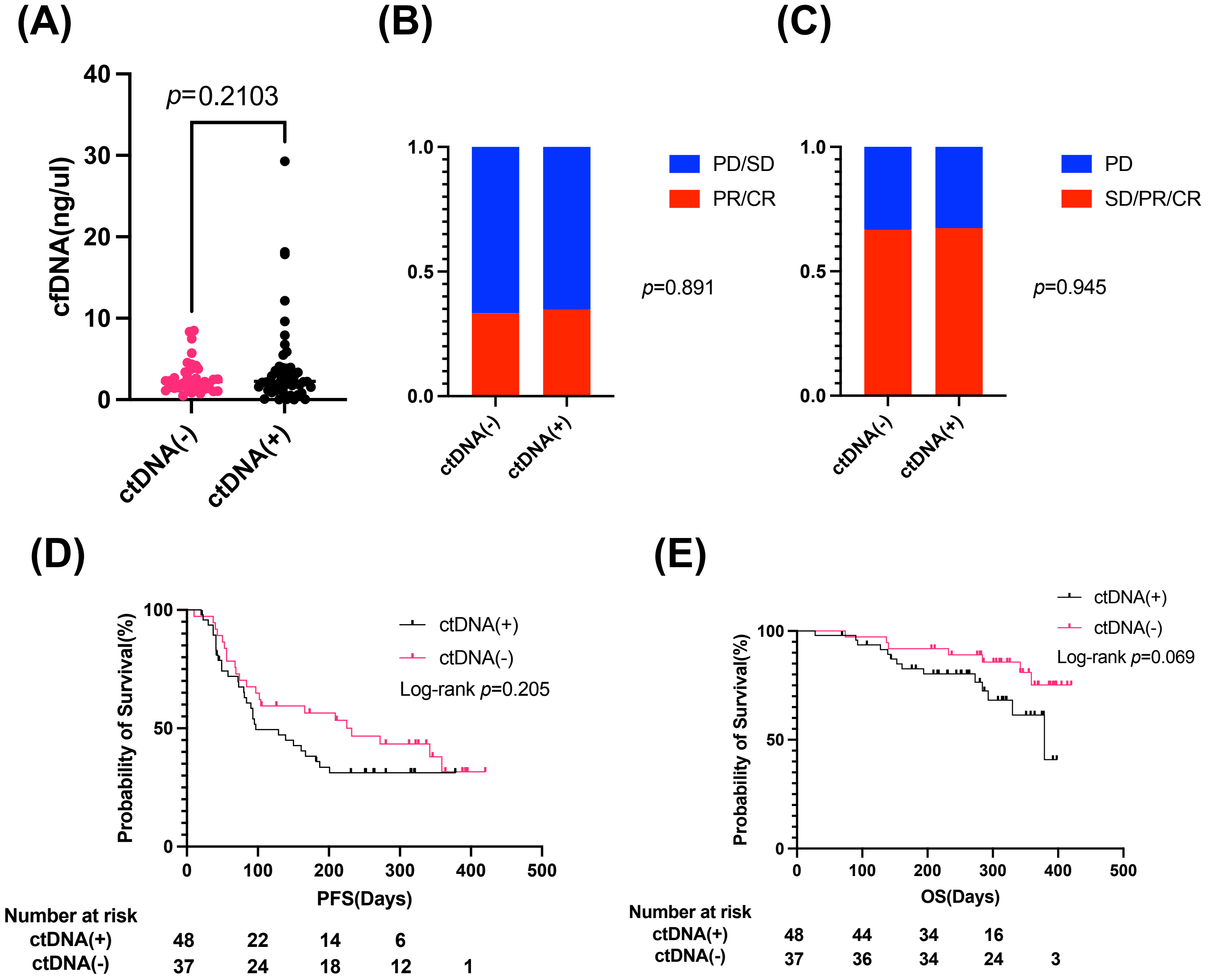

Supplementary Figure S2

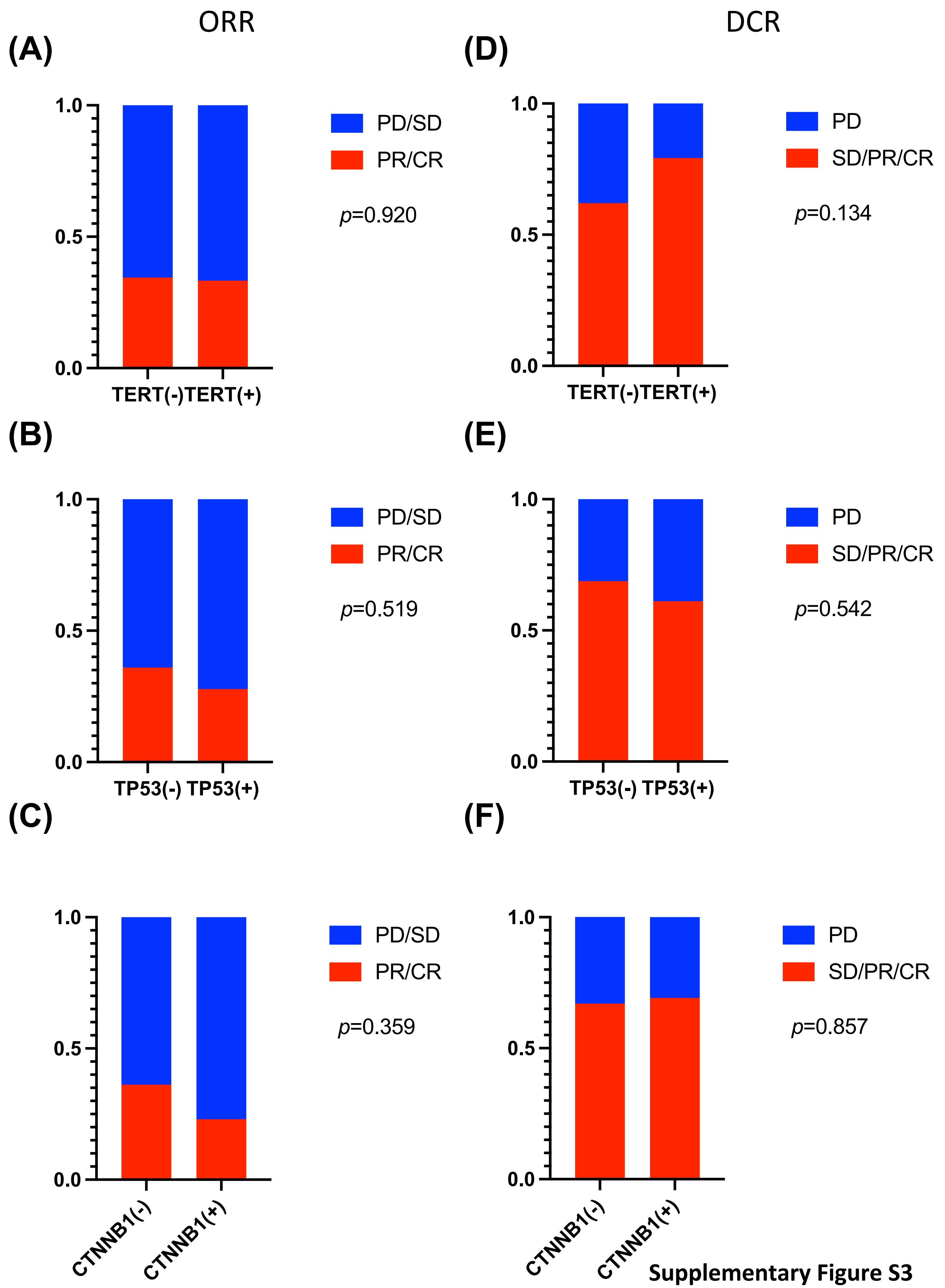

Supplementary Figure S3

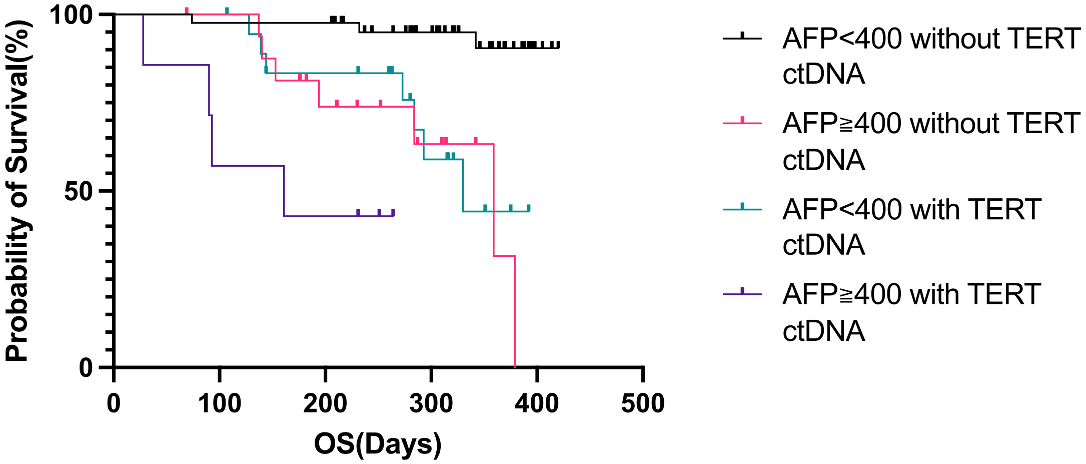

survival rate					
AFP<400 without TERT ctDNA	**100**	**98**	**98**	**95**	**90**
AFP≧400 without TERT ctDNA	**100**	**100**	**74**	**63**	
AFP<400 with TERT ctDNA	**100**	**100**	**83**	**59**	
AFP≧400 with TERT ctDNA	**100**	**57**	**43**		

Supplementary Figure S4
